# 3-Isopropyl-2-*p*-tol­yloxy-5,6,7,8-tetra­hydro-1-benzothieno[2,3-*d*]pyrimidin-4(3*H*)-one

**DOI:** 10.1107/S1600536809014962

**Published:** 2009-04-30

**Authors:** Xiao-Hua Zeng, Shou-Heng Deng, Yong-Nian Qu, Hong-Mei Wang

**Affiliations:** a Institute of Medicinal Chemistry, Yunyang Medical College, Shiyan 442000, People’s Republic of China; b Center of Oncology, People’s Hospital Affiliated with YunYang Medical College, Shi Yan 442000, People’s Republic of China; c Department of Medicinal Chemistry, Yunyang Medical College, Shiyan 442000, People’s Republic of China

## Abstract

In the title compound, C_20_H_22_N_2_O_2_S, the central thieno­pyrimidine ring system is essentially planar, with a maximum displacement of 0.023 (2) Å. The attached cyclo­hexene ring is disordered over two possible conformations, with an occupancy ratio of 0.776 (12):0.224 (12). Neither inter­molecular hydrogen-bonding inter­actions nor π–π stacking inter­actions are present in the crystal structure. The mol­ecular conformation and crystal packing are stabilized by three intra­molecular C—H⋯O hydrogen bonds and two C—H⋯π inter­actions.

## Related literature

For the biological activity of thienopyrimidin-4(3*H*)-one derivatives, see: De Laszlo *et al.* (1992*a* ▶,*b* ▶); Taguchi *et al.* (1993*a* ▶,*b* ▶); Walter (1999*a* ▶,*b* ▶,*c* ▶,*d* ▶); Walter & Zeun (2004 ▶); Ding *et al.* (2004 ▶); Santagati *et al.* (2003 ▶); Abbott GmbH Co KG (2004*a* ▶, 2004*b* ▶); Waehaelae *et al.* (2004*a* ▶,*b* ▶); Ford *et al.* (2004*a* ▶,*b* ▶); Duval *et al.* (2005 ▶). For a description of the Cambridge Structural Database, see: Allen (2002 ▶). For related structures, see: Xie *et al.* (2008 ▶); Xu *et al.* (2005 ▶); Zeng *et al.* (2005 ▶, 2006 ▶, 2007 ▶, 2008 ▶); Wang *et al.* (2006 ▶, 2007 ▶, 2008 ▶); Zheng *et al.* (2007 ▶).
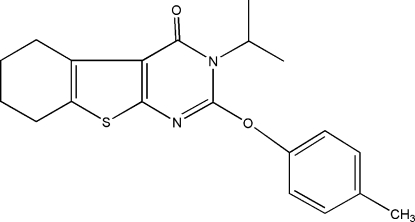


## Experimental

### 

#### Crystal data

C_20_H_22_N_2_O_2_S
*M* *_r_* = 354.47
Monoclinic, 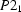

*a* = 13.2367 (7) Å
*b* = 5.7493 (3) Å
*c* = 13.4306 (7) Å
β = 115.858 (4)°
*V* = 919.76 (8) Å^3^
*Z* = 2
Mo *K*α radiation
μ = 0.19 mm^−1^
*T* = 298 K
0.20 × 0.10 × 0.10 mm

#### Data collection

Bruker SMART CCD area-detector diffractometer
Absorption correction: multi-scan (*SADABS*; Sheldrick, 1996 ▶) *T*_min_ = 0.963, *T*_max_ = 0.981
6373 measured reflections
3920 independent reflections
3370 reflections with *I* > 2σ(*I*)
*R*_int_ = 0.079

#### Refinement

*R*[*F* ^2^ > 2σ(*F* ^2^)] = 0.057
*wR*(*F* ^2^) = 0.141
*S* = 1.01
3920 reflections
248 parameters
16 restraints
H-atom parameters constrained
Δρ_max_ = 0.20 e Å^−3^
Δρ_min_ = −0.32 e Å^−3^
Absolute structure: Flack (1983 ▶), 1702 Freidel pairs
Flack parameter: 0.16 (10)

### 

Data collection: *SMART* (Bruker, 2001 ▶); cell refinement: *SAINT* (Bruker, 2001 ▶); data reduction: *SAINT* program(s) used to solve structure: *SHELXS97* (Sheldrick, 2008 ▶); program(s) used to refine structure: *SHELXL97* (Sheldrick, 2008 ▶); molecular graphics: *PLATON* (Spek, 2009 ▶); software used to prepare material for publication: *SHELXTL* (Sheldrick, 2008 ▶).

## Supplementary Material

Crystal structure: contains datablocks global, I. DOI: 10.1107/S1600536809014962/at2765sup1.cif

Structure factors: contains datablocks I. DOI: 10.1107/S1600536809014962/at2765Isup2.hkl

Additional supplementary materials:  crystallographic information; 3D view; checkCIF report

## Figures and Tables

**Table 1:** Hydrogen-bond geometry (Å, °)

*D*—H⋯*A*	*D*—H	H⋯*A*	*D*⋯*A*	*D*—H⋯*A*
C13—H13*C*⋯O2	0.96	2.41	2.959 (4)	116
C12—H12*A*⋯O2	0.96	2.31	2.871 (4)	117
C11—H11⋯O1	0.98	2.20	2.725 (3)	112
C12—H12*C*⋯*Cg*1^i^	0.96	2.94	3.838 (4)	156
C12—H12*C*⋯*Cg*2^i^	0.96	2.72	3.413 (4)	130

Symmetry code: (i) 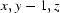. *Cg*1 and *Cg*2 are the centroids of the thio­phene (S1/C1/C6–C8) and pyrimidine (N1/N2/C7–C10) rings, respectively.

**Fig. 1.**
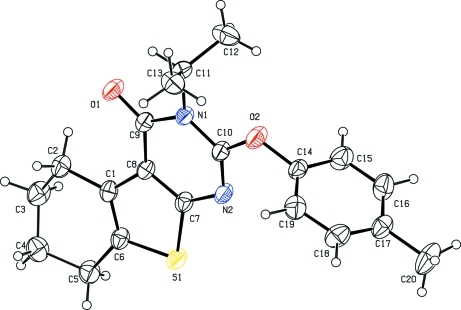
View of the molecule of showing the atom-labeling scheme. Displacement ellipsoids are drawn at 50% probability level. H-atoms are represented by circles of arbitrary size. Only the major component of the disordered cyclohexene ring is shown.

## supplementary crystallographic information

### Comment

The derivatives of heterocycles containing thienopyrimidine system, which are well known bioisosteres of quinazolines, are of great importance because of their remarkable biological properties. Some of these activities include antimicrobial or antifungal activities (De Laszlo *et al.*, 1992*a*,*b*; Walter, 1999*a*,*b*,*c*,*d*); Ding *et al.*, 2004; Walter *et al.*, 2004), significant 5-HT_1A_ and 5-HT_1B_ receptor activities (Taguchi *et al.*, 1993*a*,*b*; Abbott GmbH & Co. KG., 2004*a*,*b*), potential selective COX-2 enzyme inhibitor activity (Santagati *et al.*, 2003), 17beta-hydroxysteroid dehydrogenase inhibitor activity (Waehaelae *et al.*, 2004*a*,*b*), potassium channel inhibitor activity (Ford *et al.*, 2004*a*,*b*), and tissue transglutaminase inhibitor activity (Duval *et al.*, 2005).

In recent years, we have been engaged in the preparation of the derivatives of heterocycles *via* aza-Wittig reaction. The title compound, (I), was synthesized and structurally characterized in this context.

The molecular structure indicates that the thieno[2,3-*d*]pyrimidine moiety is a conjugated system (Fig.1). All ring atoms in thieno[2,3-*d*]pyrimidine are essentially coplanar (Xu *et al.*, 2005; Zeng *et al.*, 2005, 2006, 2007, 2008; Wang *et al.*, 2006, 2007, 2008; Zheng *et al.*, 2007; Xie *et al.*, 2008). The bond lengths and angles are within experimental error, in the ranges of values in previously reported structures in the Cambridge Structural Database (Version 5.26; Allen, 2002).

The attached cyclohexene ring is disordered. There are two possible conformations, C2—C5 and C2/C3'/C4'/C5, with an occupancy ratio of 0.77:0.23. There exists no intermolecular hydrogen bonding interaction and no π-π stacking. The molecular conformation and crystal packing are stabilized by three intramolecular C—H···O hydrogen bonds and two C—H···π interactions.

### Experimental

To a solution of iminophosphorane (1.45 g, 3 mmol) in anhydrous dichloromethane (15 ml) was added iso-propyl isocyanate (3 mmol) under dry nitrogen at room temperature. After the reaction mixture was left unstirred for 48 h at room temperature, the solvent was removed off under reduced pressure and ether/petroleum ether (1:2 *v*/*v*, 20 ml) was added to precipitate triphenylphosphine oxide. After filtration, the solvent was removed, and the residue was dissolved in CH_3_CN (15 ml). After adding 4-CH_3_—PhOH (3.1 mmol) and excess K_2_CO_3_ to the solution of carbodiimide, the mixture was stirred for 15 h at room temperature. The solution was condensed and the residue was recrystallized by EtOH to give the title compound, (I), in yield of 65% (m.p. 436 K). Elemental analysis calculated for C_20_H_22_N_2_O_2_S: C 67.77, H 6.26, N 7.90%. Found: C 67.54, H 6.32, N 7.83%. Crystals suitable for single crystal X-ray diffraction were obtained by vapor diffusion of hexane and dichloromethane (1:3 *v*/*v*) at room temperature.

### Refinement

H atoms were placed at calculated positions and treated as riding atoms, with C—H = 0.93–0.98 Å, and *U*_iso_(H) = 1.2*U*_eq_(C) or 1.5*U*_eq_(C).

### Crystal data

**Table:**

C_20_H_22_N_2_O_2_S	*F*(000) = 376
*M**_r_* = 354.47	*D*_x_ = 1.280 Mg m^−^^3^
Monoclinic, *P*2_1_	Mo *K*α radiation, λ = 0.71073 Å
Hall symbol: P 2yb	Cell parameters from 2048 reflections
*a* = 13.2367 (7) Å	θ = 2.9–24.5°
*b* = 5.7493 (3) Å	µ = 0.19 mm^−^^1^
*c* = 13.4306 (7) Å	*T* = 298 K
β = 115.858 (4)°	Block, colourless
*V* = 919.76 (8) Å^3^	0.20 × 0.10 × 0.10 mm
*Z* = 2	

### Data collection

**Table:**

Bruker SMART CCD area-detector diffractometer	3920 independent reflections
Radiation source: fine-focus sealed tube	3370 reflections with *I* > 2σ(*I*)
graphite	*R*_int_ = 0.079
φ and ω scans	θ_max_ = 27.0°, θ_min_ = 1.7°
Absorption correction: multi-scan (*SADABS*; Sheldrick, 1996)	*h* = −16→16
*T*_min_ = 0.963, *T*_max_ = 0.981	*k* = −7→7
6373 measured reflections	*l* = −17→15

### Refinement

**Table:**

Refinement on *F*^2^	Secondary atom site location: difference Fourier map
Least-squares matrix: full	Hydrogen site location: inferred from neighbouring sites
*R*[*F*^2^ > 2σ(*F*^2^)] = 0.057	H-atom parameters constrained
*wR*(*F*^2^) = 0.141	*w* = 1/[σ^2^(*F*_o_^2^) + (0.0798*P*)^2^] where *P* = (*F*_o_^2^ + 2*F*_c_^2^)/3
*S* = 1.01	(Δ/σ)_max_ < 0.001
3920 reflections	Δρ_max_ = 0.20 e Å^−^^3^
248 parameters	Δρ_min_ = −0.32 e Å^−^^3^
16 restraints	Absolute structure: Flack (1983), 1702 Freidel pairs
Primary atom site location: structure-invariant direct methods	Flack parameter: 0.16 (10)

### Special details

**Table:**

Geometry. All e.s.d.'s (except the e.s.d. in the dihedral angle between two l.s. planes) are estimated using the full covariance matrix. The cell e.s.d.'s are taken into account individually in the estimation of e.s.d.'s in distances, angles and torsion angles; correlations between e.s.d.'s in cell parameters are only used when they are defined by crystal symmetry. An approximate (isotropic) treatment of cell e.s.d.'s is used for estimating e.s.d.'s involving l.s. planes.
Refinement. Refinement of *F*^2^ against ALL reflections. The weighted *R*-factor *wR* and goodness of fit *S* are based on *F*^2^, conventional *R*-factors *R* are based on *F*, with *F* set to zero for negative *F*^2^. The threshold expression of *F*^2^ > σ(*F*^2^) is used only for calculating *R*-factors(gt) *etc*. and is not relevant to the choice of reflections for refinement. *R*-factors based on *F*^2^ are statistically about twice as large as those based on *F*, and *R*- factors based on ALL data will be even larger.

### Fractional atomic coordinates and isotropic or equivalent isotropic displacement parameters (Å^2^)

**Table:**

	*x*	*y*	*z*	*U*_iso_*/*U*_eq_	Occ. (<1)
C1	0.20878 (19)	0.6656 (5)	0.88050 (19)	0.0448 (6)	
C2	0.0948 (2)	0.6877 (6)	0.8794 (2)	0.0563 (7)	
H2A	0.0446	0.7700	0.8132	0.068*	0.776 (12)
H2B	0.0639	0.5339	0.8776	0.068*	0.776 (12)
H2C	0.0817	0.5593	0.9191	0.068*	0.224 (12)
H2D	0.0370	0.6849	0.8037	0.068*	0.224 (12)
C3	0.1018 (4)	0.8162 (11)	0.9796 (5)	0.0662 (16)	0.776 (12)
H3A	0.1348	0.7157	1.0439	0.079*	0.776 (12)
H3B	0.0268	0.8576	0.9693	0.079*	0.776 (12)
C4	0.1717 (4)	1.0336 (10)	0.9996 (6)	0.0690 (17)	0.776 (12)
H4A	0.1703	1.1175	1.0616	0.083*	0.776 (12)
H4B	0.1383	1.1329	0.9349	0.083*	0.776 (12)
C3'	0.0904 (12)	0.916 (2)	0.9334 (14)	0.061 (5)	0.224 (12)
H3'1	0.0829	1.0405	0.8817	0.073*	0.224 (12)
H3'2	0.0230	0.9169	0.9448	0.073*	0.224 (12)
C4'	0.1882 (9)	0.974 (5)	1.0420 (11)	0.068 (6)	0.224 (12)
H4'1	0.1965	0.8556	1.0964	0.081*	0.224 (12)
H4'2	0.1758	1.1224	1.0692	0.081*	0.224 (12)
C5	0.2932 (2)	0.9853 (6)	1.0236 (3)	0.0615 (8)	
H5A	0.3283	1.1265	1.0144	0.074*	0.776 (12)
H5B	0.3346	0.9322	1.0993	0.074*	0.776 (12)
H5C	0.2996	1.1376	0.9958	0.074*	0.224 (12)
H5D	0.3578	0.9636	1.0943	0.074*	0.224 (12)
C6	0.2953 (2)	0.8022 (5)	0.9451 (2)	0.0489 (6)	
C7	0.3471 (2)	0.5369 (5)	0.8298 (2)	0.0457 (6)	
C8	0.23832 (18)	0.5102 (5)	0.81357 (19)	0.0419 (5)	
C9	0.1665 (2)	0.3547 (5)	0.7278 (2)	0.0440 (5)	
C10	0.33155 (19)	0.2882 (5)	0.6970 (2)	0.0472 (6)	
C11	0.1476 (2)	0.1089 (5)	0.5680 (2)	0.0476 (6)	
H11	0.0727	0.1104	0.5658	0.057*	
C12	0.1818 (3)	−0.1432 (6)	0.5773 (3)	0.0763 (10)	
H12A	0.2536	−0.1556	0.5760	0.114*	
H12B	0.1269	−0.2287	0.5163	0.114*	
H12C	0.1867	−0.2060	0.6455	0.114*	
C13	0.1348 (3)	0.2214 (6)	0.4619 (2)	0.0640 (8)	
H13A	0.1085	0.3781	0.4587	0.096*	
H13B	0.0817	0.1349	0.4001	0.096*	
H13C	0.2061	0.2229	0.4591	0.096*	
C14	0.4833 (2)	0.1798 (5)	0.6585 (2)	0.0515 (7)	
C15	0.5521 (3)	0.0051 (7)	0.7178 (3)	0.0704 (9)	
H15	0.5252	−0.1141	0.7466	0.084*	
C16	0.6628 (3)	0.0076 (7)	0.7348 (3)	0.0743 (9)	
H16	0.7098	−0.1137	0.7741	0.089*	
C17	0.7055 (2)	0.1829 (6)	0.6956 (2)	0.0592 (8)	
C18	0.6321 (3)	0.3536 (7)	0.6344 (3)	0.0721 (9)	
H18	0.6583	0.4727	0.6050	0.086*	
C19	0.5207 (3)	0.3543 (6)	0.6149 (3)	0.0710 (9)	
H19	0.4724	0.4715	0.5730	0.085*	
C20	0.8275 (2)	0.1872 (10)	0.7183 (3)	0.0936 (15)	
H20A	0.8639	0.3178	0.7646	0.140*	
H20B	0.8332	0.2006	0.6496	0.140*	
H20C	0.8632	0.0460	0.7549	0.140*	
N1	0.22102 (15)	0.2496 (4)	0.66777 (15)	0.0435 (4)	
N2	0.39874 (17)	0.4226 (4)	0.77485 (19)	0.0524 (6)	
O2	0.36853 (15)	0.1676 (4)	0.63340 (17)	0.0689 (7)	
O1	0.06852 (14)	0.3137 (4)	0.70296 (16)	0.0607 (6)	
S1	0.41623 (5)	0.74703 (15)	0.92707 (6)	0.0585 (2)	

### Atomic displacement parameters (Å^2^)

**Table:**

	*U*^11^	*U*^22^	*U*^33^	*U*^12^	*U*^13^	*U*^23^
C1	0.0409 (13)	0.0542 (15)	0.0392 (12)	0.0072 (10)	0.0172 (10)	0.0072 (11)
C2	0.0453 (14)	0.068 (2)	0.0600 (16)	0.0029 (12)	0.0267 (12)	−0.0012 (14)
C3	0.062 (3)	0.086 (4)	0.062 (3)	−0.003 (2)	0.038 (2)	−0.010 (3)
C4	0.075 (3)	0.074 (4)	0.070 (4)	0.005 (2)	0.042 (3)	−0.014 (3)
C3'	0.053 (7)	0.072 (8)	0.060 (8)	0.002 (6)	0.026 (6)	0.000 (6)
C4'	0.075 (9)	0.081 (10)	0.056 (9)	0.012 (6)	0.036 (7)	0.012 (7)
C5	0.0554 (16)	0.071 (2)	0.0553 (16)	0.0041 (14)	0.0213 (13)	−0.0128 (15)
C6	0.0409 (12)	0.0616 (17)	0.0410 (12)	0.0051 (11)	0.0149 (10)	−0.0014 (12)
C7	0.0371 (13)	0.0556 (15)	0.0383 (12)	0.0036 (11)	0.0107 (10)	−0.0022 (11)
C8	0.0347 (11)	0.0508 (13)	0.0373 (12)	0.0014 (10)	0.0130 (10)	0.0026 (10)
C9	0.0369 (12)	0.0502 (14)	0.0449 (13)	0.0016 (10)	0.0179 (10)	0.0058 (11)
C10	0.0360 (11)	0.0563 (16)	0.0490 (13)	0.0013 (11)	0.0182 (10)	−0.0032 (12)
C11	0.0381 (13)	0.0495 (15)	0.0485 (14)	−0.0052 (11)	0.0127 (11)	−0.0059 (12)
C12	0.095 (3)	0.0463 (17)	0.072 (2)	−0.0096 (16)	0.0220 (19)	0.0016 (16)
C13	0.0728 (18)	0.0565 (18)	0.0483 (15)	0.0039 (15)	0.0130 (13)	−0.0016 (14)
C14	0.0381 (13)	0.0631 (18)	0.0561 (15)	−0.0044 (11)	0.0232 (12)	−0.0184 (13)
C15	0.0602 (18)	0.075 (2)	0.082 (2)	−0.0034 (17)	0.0366 (17)	0.0130 (19)
C16	0.0570 (18)	0.083 (2)	0.077 (2)	0.0165 (17)	0.0243 (16)	0.0157 (19)
C17	0.0473 (14)	0.085 (2)	0.0514 (15)	−0.0026 (14)	0.0276 (12)	−0.0128 (15)
C18	0.0622 (19)	0.085 (2)	0.076 (2)	−0.0121 (18)	0.0372 (17)	0.0070 (18)
C19	0.0608 (18)	0.067 (2)	0.083 (2)	0.0070 (16)	0.0292 (17)	0.0075 (18)
C20	0.0496 (17)	0.166 (5)	0.073 (2)	0.002 (2)	0.0332 (16)	−0.020 (3)
N1	0.0338 (9)	0.0498 (11)	0.0439 (10)	−0.0019 (10)	0.0143 (8)	−0.0033 (10)
N2	0.0319 (10)	0.0662 (15)	0.0566 (13)	−0.0020 (10)	0.0169 (9)	−0.0152 (11)
O2	0.0398 (10)	0.0937 (17)	0.0744 (13)	−0.0097 (10)	0.0261 (9)	−0.0374 (12)
O1	0.0361 (9)	0.0808 (15)	0.0670 (11)	−0.0103 (9)	0.0242 (8)	−0.0142 (11)
S1	0.0359 (3)	0.0722 (5)	0.0603 (4)	−0.0040 (3)	0.0144 (3)	−0.0216 (4)

### Geometric parameters (Å, °)

**Table:**

C1—C6	1.347 (4)	C9—O1	1.213 (3)
C1—C8	1.437 (4)	C9—N1	1.429 (3)
C1—C2	1.508 (3)	C10—N2	1.292 (3)
C2—C3	1.502 (5)	C10—O2	1.348 (3)
C2—C3'	1.515 (10)	C10—N1	1.358 (3)
C2—H2A	0.9700	C11—N1	1.503 (3)
C2—H2B	0.9700	C11—C13	1.506 (4)
C2—H2C	0.9700	C11—C12	1.508 (5)
C2—H2D	0.9700	C11—H11	0.9800
C3—C4	1.508 (7)	C12—H12A	0.9600
C3—H3A	0.9700	C12—H12B	0.9600
C3—H3B	0.9700	C12—H12C	0.9600
C4—C5	1.521 (5)	C13—H13A	0.9600
C4—H4A	0.9700	C13—H13B	0.9600
C4—H4B	0.9700	C13—H13C	0.9600
C3'—C4'	1.505 (10)	C14—C15	1.356 (5)
C3'—H3'1	0.9700	C14—C19	1.359 (4)
C3'—H3'2	0.9700	C14—O2	1.407 (3)
C4'—C5	1.515 (9)	C15—C16	1.382 (4)
C4'—H4'1	0.9700	C15—H15	0.9300
C4'—H4'2	0.9700	C16—C17	1.368 (5)
C5—C6	1.499 (4)	C16—H16	0.9300
C5—H5A	0.9700	C17—C18	1.373 (5)
C5—H5B	0.9700	C17—C20	1.508 (4)
C5—H5C	0.9700	C18—C19	1.381 (4)
C5—H5D	0.9700	C18—H18	0.9300
C6—S1	1.749 (3)	C19—H19	0.9300
C7—C8	1.368 (3)	C20—H20A	0.9600
C7—N2	1.372 (3)	C20—H20B	0.9600
C7—S1	1.721 (3)	C20—H20C	0.9600
C8—C9	1.440 (4)		
			
C6—C1—C8	112.2 (2)	H5A—C5—H5D	88.0
C6—C1—C2	121.6 (2)	H5B—C5—H5D	23.0
C8—C1—C2	126.2 (2)	H5C—C5—H5D	107.8
C3—C2—C1	111.2 (3)	C1—C6—C5	126.2 (2)
C3—C2—C3'	31.2 (6)	C1—C6—S1	112.19 (19)
C1—C2—C3'	108.4 (7)	C5—C6—S1	121.6 (2)
C3—C2—H2A	109.4	C8—C7—N2	126.7 (2)
C1—C2—H2A	109.4	C8—C7—S1	111.93 (19)
C3'—C2—H2A	82.1	N2—C7—S1	121.34 (18)
C3—C2—H2B	109.4	C7—C8—C1	112.7 (2)
C1—C2—H2B	109.4	C7—C8—C9	118.6 (2)
C3'—C2—H2B	134.4	C1—C8—C9	128.4 (2)
H2A—C2—H2B	108.0	O1—C9—N1	120.3 (2)
C3—C2—H2C	80.7	O1—C9—C8	126.4 (2)
C1—C2—H2C	110.4	N1—C9—C8	113.2 (2)
C3'—C2—H2C	110.1	N2—C10—O2	120.6 (2)
H2A—C2—H2C	131.4	N2—C10—N1	127.4 (2)
H2B—C2—H2C	31.3	O2—C10—N1	112.1 (2)
C3—C2—H2D	131.3	N1—C11—C13	112.0 (2)
C1—C2—H2D	109.7	N1—C11—C12	112.9 (2)
C3'—C2—H2D	109.9	C13—C11—C12	113.4 (3)
H2A—C2—H2D	30.2	N1—C11—H11	105.9
H2B—C2—H2D	80.0	C13—C11—H11	105.9
H2C—C2—H2D	108.4	C12—C11—H11	105.9
C2—C3—C4	111.1 (5)	C11—C12—H12A	109.5
C2—C3—H3A	109.4	C11—C12—H12B	109.5
C4—C3—H3A	109.4	H12A—C12—H12B	109.5
C2—C3—H3B	109.4	C11—C12—H12C	109.5
C4—C3—H3B	109.4	H12A—C12—H12C	109.5
H3A—C3—H3B	108.0	H12B—C12—H12C	109.5
C3—C4—C5	113.3 (4)	C11—C13—H13A	109.5
C3—C4—H4A	108.9	C11—C13—H13B	109.5
C5—C4—H4A	108.9	H13A—C13—H13B	109.5
C3—C4—H4B	108.9	C11—C13—H13C	109.5
C5—C4—H4B	108.9	H13A—C13—H13C	109.5
H4A—C4—H4B	107.7	H13B—C13—H13C	109.5
C4'—C3'—C2	117.3 (15)	C15—C14—C19	121.5 (3)
C4'—C3'—H3'1	108.0	C15—C14—O2	118.8 (3)
C2—C3'—H3'1	108.0	C19—C14—O2	119.4 (3)
C4'—C3'—H3'2	108.0	C14—C15—C16	118.8 (3)
C2—C3'—H3'2	108.0	C14—C15—H15	120.6
H3'1—C3'—H3'2	107.2	C16—C15—H15	120.6
C3'—C4'—C5	108.3 (10)	C17—C16—C15	122.0 (3)
C3'—C4'—H4'1	110.0	C17—C16—H16	119.0
C5—C4'—H4'1	110.0	C15—C16—H16	119.0
C3'—C4'—H4'2	110.0	C16—C17—C18	117.0 (3)
C5—C4'—H4'2	110.0	C16—C17—C20	121.3 (3)
H4'1—C4'—H4'2	108.4	C18—C17—C20	121.6 (3)
C6—C5—C4'	112.6 (9)	C17—C18—C19	122.2 (3)
C6—C5—C4	108.8 (3)	C17—C18—H18	118.9
C4'—C5—C4	23.5 (6)	C19—C18—H18	118.9
C6—C5—H5A	109.9	C14—C19—C18	118.4 (3)
C4'—C5—H5A	125.5	C14—C19—H19	120.8
C4—C5—H5A	109.9	C18—C19—H19	120.8
C6—C5—H5B	109.9	C17—C20—H20A	109.5
C4'—C5—H5B	87.4	C17—C20—H20B	109.5
C4—C5—H5B	109.9	H20A—C20—H20B	109.5
H5A—C5—H5B	108.3	C17—C20—H20C	109.5
C6—C5—H5C	109.4	H20A—C20—H20C	109.5
C4'—C5—H5C	110.0	H20B—C20—H20C	109.5
C4—C5—H5C	90.9	C10—N1—C9	120.8 (2)
H5A—C5—H5C	21.3	C10—N1—C11	122.9 (2)
H5B—C5—H5C	125.7	C9—N1—C11	116.21 (18)
C6—C5—H5D	108.6	C10—N2—C7	113.1 (2)
C4'—C5—H5D	108.3	C10—O2—C14	118.4 (2)
C4—C5—H5D	129.0	C7—S1—C6	90.97 (12)
			
C6—C1—C2—C3	−17.9 (5)	C19—C14—C15—C16	0.6 (5)
C8—C1—C2—C3	164.4 (4)	O2—C14—C15—C16	174.8 (3)
C6—C1—C2—C3'	15.2 (8)	C14—C15—C16—C17	1.4 (5)
C8—C1—C2—C3'	−162.5 (8)	C15—C16—C17—C18	−2.5 (5)
C1—C2—C3—C4	46.7 (6)	C15—C16—C17—C20	177.8 (3)
C3'—C2—C3—C4	−44.0 (11)	C16—C17—C18—C19	1.7 (5)
C2—C3—C4—C5	−62.5 (8)	C20—C17—C18—C19	−178.6 (3)
C3—C2—C3'—C4'	53.3 (13)	C15—C14—C19—C18	−1.4 (5)
C1—C2—C3'—C4'	−47.5 (19)	O2—C14—C19—C18	−175.5 (3)
C2—C3'—C4'—C5	61 (3)	C17—C18—C19—C14	0.2 (5)
C3'—C4'—C5—C6	−39 (2)	N2—C10—N1—C9	−2.7 (4)
C3'—C4'—C5—C4	46.5 (12)	O2—C10—N1—C9	177.7 (2)
C3—C4—C5—C6	43.0 (7)	N2—C10—N1—C11	173.4 (3)
C3—C4—C5—C4'	−61 (2)	O2—C10—N1—C11	−6.3 (4)
C8—C1—C6—C5	179.2 (3)	O1—C9—N1—C10	−177.9 (3)
C2—C1—C6—C5	1.2 (4)	C8—C9—N1—C10	3.8 (3)
C8—C1—C6—S1	0.0 (3)	O1—C9—N1—C11	5.8 (4)
C2—C1—C6—S1	−178.0 (2)	C8—C9—N1—C11	−172.6 (2)
C4'—C5—C6—C1	11.4 (9)	C13—C11—N1—C10	−64.3 (3)
C4—C5—C6—C1	−13.4 (5)	C12—C11—N1—C10	65.2 (3)
C4'—C5—C6—S1	−169.5 (9)	C13—C11—N1—C9	112.0 (3)
C4—C5—C6—S1	165.8 (3)	C12—C11—N1—C9	−118.6 (3)
N2—C7—C8—C1	−176.8 (3)	O2—C10—N2—C7	178.4 (3)
S1—C7—C8—C1	0.7 (3)	N1—C10—N2—C7	−1.2 (4)
N2—C7—C8—C9	−2.7 (4)	C8—C7—N2—C10	4.0 (4)
S1—C7—C8—C9	174.81 (19)	S1—C7—N2—C10	−173.3 (2)
C6—C1—C8—C7	−0.4 (3)	N2—C10—O2—C14	4.1 (4)
C2—C1—C8—C7	177.4 (2)	N1—C10—O2—C14	−176.3 (2)
C6—C1—C8—C9	−173.8 (2)	C15—C14—O2—C10	98.6 (3)
C2—C1—C8—C9	4.1 (4)	C19—C14—O2—C10	−87.0 (3)
C7—C8—C9—O1	−179.5 (3)	C8—C7—S1—C6	−0.6 (2)
C1—C8—C9—O1	−6.5 (4)	N2—C7—S1—C6	177.0 (2)
C7—C8—C9—N1	−1.3 (3)	C1—C6—S1—C7	0.4 (2)
C1—C8—C9—N1	171.7 (2)	C5—C6—S1—C7	−178.9 (2)

### Hydrogen-bond geometry (Å, °)

**Table:**

*D*—H···*A*	*D*—H	H···*A*	*D*···*A*	*D*—H···*A*
C13—H13C···O2	0.96	2.41	2.959 (4)	116
C12—H12A···O2	0.96	2.31	2.871 (4)	117
C11—H11···O1	0.98	2.20	2.725 (3)	112
C12—H12C···Cg1^i^	0.96	2.94	3.838 (4)	156
C12—H12C···Cg2^i^	0.96	2.72	3.413 (4)	130

Symmetry codes: (i) *x*, *y*−1, *z*.
